# T-type Ca^2+^ channels are required for enhanced sympathetic axon growth by TNFα reverse signalling

**DOI:** 10.1098/rsob.160288

**Published:** 2017-01-18

**Authors:** Lilian Kisiswa, Clara Erice, Laurent Ferron, Sean Wyatt, Catarina Osório, Annette C. Dolphin, Alun M. Davies

**Affiliations:** 1 School Biosciences, Cardiff University, Museum Avenue, Cardiff CF10 3AX, UK; 2 Department of Neuroscience, Physiology and Pharmacology, University College London, Andrew Huxley Building, Gower Street, London WC1E 6BT, UK

**Keywords:** development, reverse signalling, sympathetic neuron, axon growth, TNFα, Ca^2+^ channels

## Abstract

Tumour necrosis factor receptor 1 (TNFR1)-activated TNFα reverse signalling, in which membrane-integrated TNFα functions as a receptor for TNFR1, enhances axon growth from developing sympathetic neurons and plays a crucial role in establishing sympathetic innervation. Here, we have investigated the link between TNFα reverse signalling and axon growth in cultured sympathetic neurons. TNFR1-activated TNFα reverse signalling promotes Ca^2+^ influx, and highly selective T-type Ca^2+^ channel inhibitors, but not pharmacological inhibitors of L-type, N-type and P/Q-type Ca^2+^ channels, prevented enhanced axon growth. T-type Ca^2+^ channel-specific inhibitors eliminated Ca^2+^ spikes promoted by TNFα reverse signalling in axons and prevented enhanced axon growth when applied locally to axons, but not when applied to cell somata. Blocking action potential generation did not affect the effect of TNFα reverse signalling on axon growth, suggesting that propagated action potentials are not required for enhanced axon growth. TNFα reverse signalling enhanced protein kinase C (PKC) activation, and pharmacological inhibition of PKC prevented the axon growth response. These results suggest that TNFα reverse signalling promotes opening of T-type Ca^2+^ channels along sympathetic axons, which is required for enhanced axon growth.

## 1. Introduction

A variety of extracellular signals participate in regulating the establishment of sympathetic innervation in the developing peripheral nervous system [1,2]. The most extensively studied and best understood of these is nerve growth factor (NGF), a secreted protein synthesized in tissues innervated by NGF-responsive neurons. NGF promotes the survival of developing sympathetic neurons, and level of NGF synthesis in different tissues regulates the number of neurons that innervate these tissues by restricting the extent of cell death among the innervating neurons during development [3]. NGF also acts on sympathetic axon terminals within target tissues to promote growth and branching [4]. Whereas retrograde PI3-kinase/Akt signalling to the cell body plays an important role in mediating NGF-promoted survival, activation of ERK1/ERK2 downstream of TrkA, the NGF receptor tyrosine kinase, plays a major role in mediating the local axon growth-promoting effects of NGF [5–8].

A recently discovered target-derived signal that also promotes sympathetic axon growth and branching during the stage in development when sympathetic axons are ramifying in their targets is tumour necrosis factor receptor 1 (TNFR1) [9]. TNFR1 expressed by sympathetic target tissues acts as a ligand for membrane-integrated TNFα expressed along sympathetic axons, and TNFR1-activated TNFα reverse signalling enhances sympathetic axon growth and branching. Mice lacking either TNFα or TNFR1 display greatly reduced sympathetic innervation density in multiple tissues, but unlike NGF-deficient mice, these mice show no deficits in sympathetic neuron number. As with NGF, activation of ERK1/ERK2 signalling plays a key role in mediating the axon growth-promoting effects of TNFα reverse signalling. Activation of ERK1/ERK2 by TNFα reverse signalling is due to rapid Ca^2+^ influx [9]; however, the identity of the channels that open in response to TNFα reverse signalling is not known. Our aim here was to identify these channels, determine where on sympathetic neurons they are functionally relevant for axon growth in response to TNFR1-activated TNFα reverse signalling, and provide a link between Ca^2+^ influx and ERK1/ERK2 activation. Using a combination of pharmacological studies, electrophysiology, Ca^2+^ reporter studies and western blot analysis, we show that activation of TNFα reverse signalling in sympathetic axons increases T-type Ca^2+^ channel activation and the subsequent activation of protein kinase C (PKC) and ERK1/ERK2 are required for enhanced axon growth.

## 2. Results

### 2.1. T-type Ca^2+^ channels are required for TNFR1-Fc-promoted axon growth

**Figure 1.**
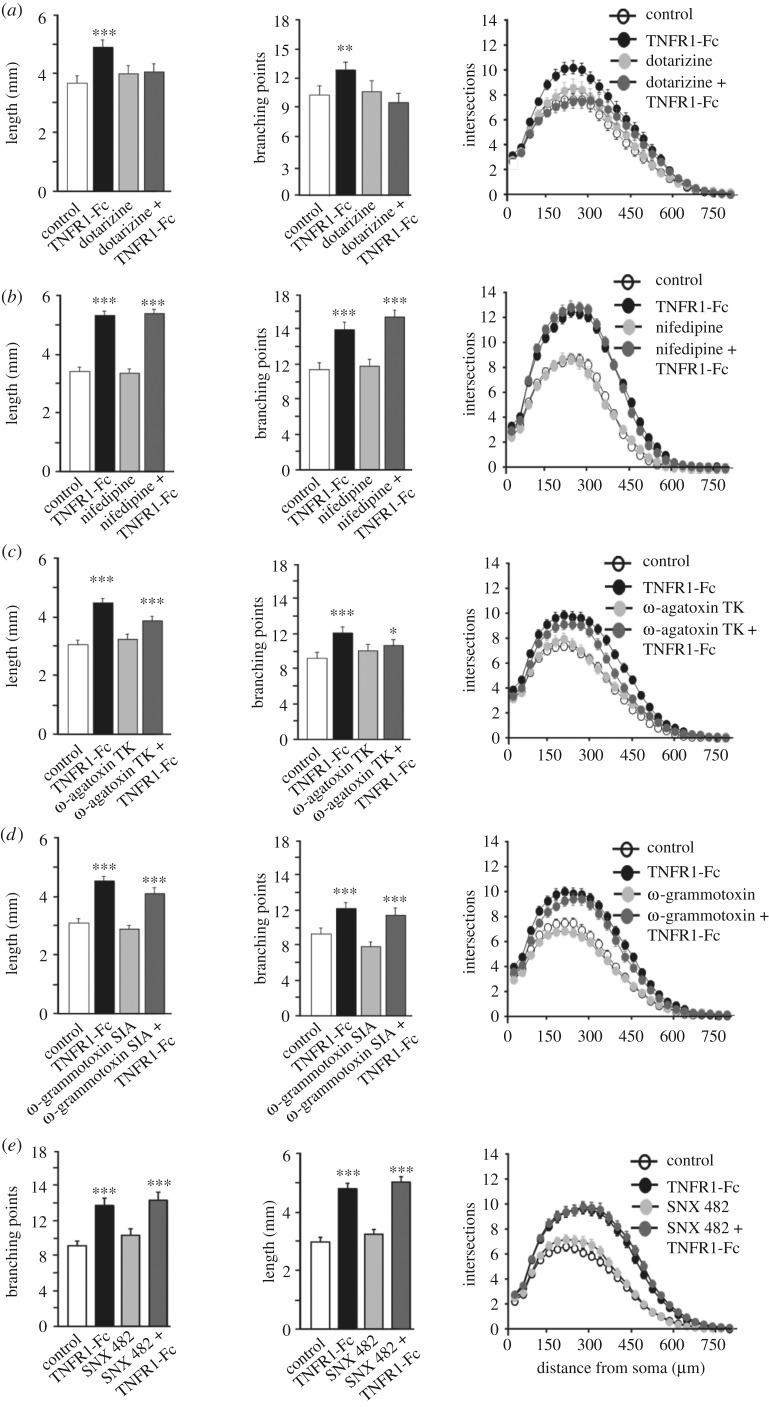
Voltage-sensitive Ca^2+^ channels other than L-, N-, P/Q- and R-type are required for TNFR1-Fc-promoted neurite growth. Bar charts of length and branch point number and the Sholl profiles of P0 SCG neurons cultured for 24 h with either 10 ng ml^−1^ NGF alone (control), NGF and 10 ng ml^−1^ TNFR1-Fc, NGF and calcium channel blocker or NGF, TNFR1-Fc and calcium channel blocker. Data from experimental series using the blockers as indicated: 1 µM dotarizine, 10 µM nifedipine, 100 nM ω-agatoxin TK, 10 nM ω-grammotoxin SIA and 60 nM SNX 482. The mean ± s.e.m. of neurite arbour data of at least 150 neurons per condition combined from three separate experiments of each type are shown (***p < 0.001, **p < 0.01, *p < 0.05, statistical comparison with control).

Our demonstration that activation of TNFα reverse signalling causes Ca^2+^ influx in postnatal sympathetic neurons and that this is necessary for enhanced axon growth [9] implicates ligand or voltage-gated Ca^2+^ channels in the axon growth response to TNFR1. To examine this, we first tested whether the broad-spectrum voltage-gated Ca^2+^ channel blocker dotarizine [10,11] inhibits the growth response to TNFα reverse signalling. Low density dissociated cultures of superior cervical ganglion (SCG) neurons were plated in medium containing NGF to sustain their survival and were treated with a divalent TNFR1-Fc chimera to activate TNFα reverse signalling [9] in the presence and absence of dotarizine. After 24 h, quantification of the size and complexity of the neurite arbours showed that the TNFR1-Fc chimera caused highly significant increases in neurite length and branch point number (figure 1). Accordingly, the Sholl profiles, which plot neurite intersections with a series of concentric circles centred on the cell body, were clearly larger and more complex than those for neurons grown with NGF alone (control cultures). The size and complexity of the neurite arbours of neurons treated with dotarizine alone were not significantly different from those in control cultures. However, 1 µM dotarizine completely prevented TNFR1-Fc enhanced neurite growth (figure 1). These studies suggest that voltage-gated Ca^2+^ channels at the plasma membrane are required for the axon growth enhancing effect of TNFα reverse signalling.

**Figure 2.**
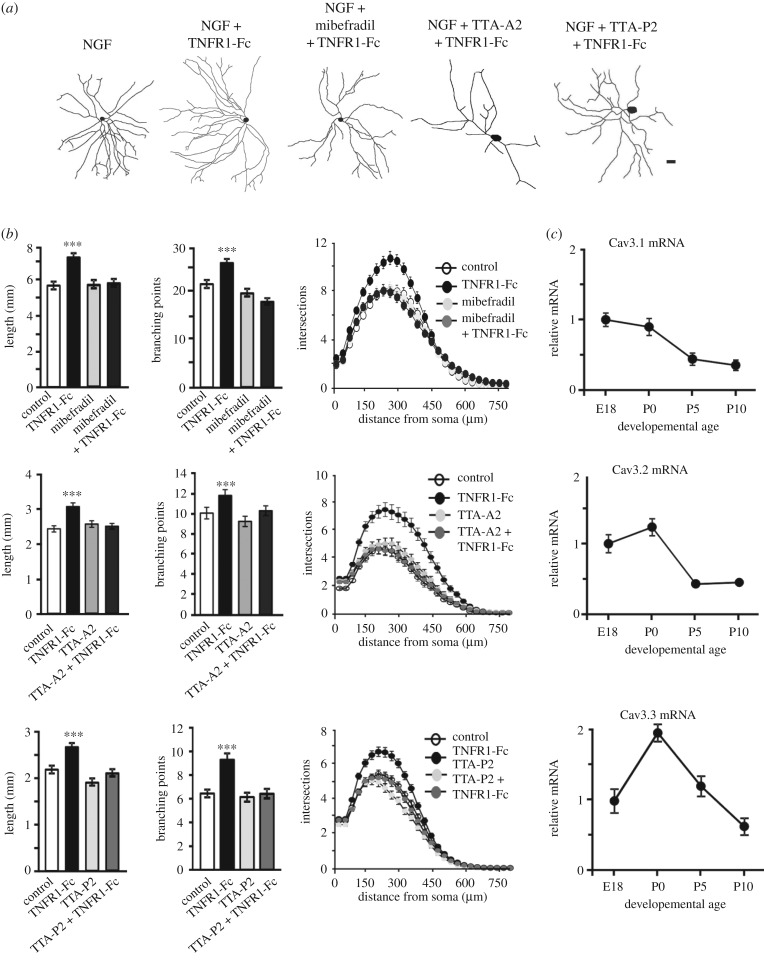
Blocking T-type Ca^2+^ channels inhibits TNFR1-Fc-promoted neurite growth. (*a*) Representative camera lucida drawings of P0 SCG neurons cultured for 24 h with either 10 ng ml^−1^ NGF alone, NGF and 10 ng ml^−1^ TNFR1-Fc or NGF, TNFR1-Fc and a T-type Ca^2+^ channel blocker (1 µM mibefradil, 200 nM TTA-A2 and 200 nM TTA-P2). Scale bar, 100 µm. (*b*) Bar charts of length and branch point number and the Sholl profiles of P0 SCG neurons cultured for 24 h with either NGF alone (control), NGF plus TNFR1-Fc, NGF plus a T-type Ca^2+^ channel blocker (as indicated) or NGF, TNFR1-Fc and a T-type Ca^2+^ channel blocker. Mean ± s.e.m. of neurite arbour data of at least 150 neurons per condition combined from three separate experiments of each type, ***p < 0.001, **p < 0.01, *p < 0.05, statistical comparison with control. (*c*) Levels of Ca_v_3.1, Ca_v_3.2 and Ca_v_3.3 mRNAs relative to reference mRNAs in SCG of different ages. The data are normalized to a value of 1.0 at the E18 data. Mean ± s.e.m. of data from four separate sets of ganglia at each age are shown.

To ascertain which kinds of voltage-gated Ca^2+^ channels are required for the enhanced axon growth response to TNFα reverse signalling, we carried out similar studies using subtype-selective Ca^2+^ channel blockers [12]. For these experiments, we used 10 µM nifedipine which is a selective blocker of L-type Ca^2+^ channels [13], 100 nM ω-agatoxin TK which blocks P/Q-type Ca^2+^ channels, [14], 10 nM ω-grammotoxin SIA which blocks N-type and P/Q-type Ca^2+^ channels [15,16], 60 nM SNX 482 which blocks R-type Ca^2+^ channels [17] and several T-type Ca^2+^ channel blockers, 1 µM mibefradil [18–20], 200 nM TTA-A2 [21] and 200 nM TTA-P2 [22]. None of these Ca^2+^ channel inhibitors had any significant effect on the extent of NGF-promoted axon growth from P0 SCG neurons when added in the absence of TNFR1-Fc (figures 1 and 2). None of the blockers nifedipine, ω-agatoxin TK, ω-grammotoxin SIA or SNX 482 significantly affected TNFR1-Fc-enhanced axon growth (figure 1), suggesting that none of the L-type, N-type, P/Q-type or R-type Ca^2+^ channels are required for the enhanced axon growth response to TNFR1-Fc. However, each of the T-type Ca^2+^ channel blockers (mibefradil, TTA-A2 and TTA-P2) completely inhibited TNFR1-Fc-enhanced axon growth (figure 2*a,b*). None of these T-type Ca^2+^ channel blockers significantly affected neuronal survival either alone or in the presence of TNFR1-Fc at the concentrations used (not shown). These findings suggest that T-type Ca^2+^ channels are required for TNFR1-enhanced axon growth.

### 2.2. Expression of T-type Ca^2+^ channel mRNA in developing superior cervical ganglion neurons

TNFα reverse signalling enhances axon growth from SCG neurons during a narrow developmental window between P0 and P5 when sympathetic axons are ramifying extensively in their targets [9]. To confirm expression of transcripts encoding T-type Ca^2+^ channels in SCG during this period of development, we used real-time PCR to quantify the levels of mRNAs encoding these channels in SCG dissected from mice at stages from E18 to P10. Separate genes, Ca_v_3.1, Ca_v_3.2 and Ca_v_3.3, encode three T-type Ca^2+^ channel isoforms that are all low voltage-activated and inactivating, but differ slightly in their biophysical properties and distribution [23]. Real-time PCR revealed that transcripts for all three genes are expressed throughout this period of development (figure 2*c*). There was an overall decrease in level of Ca_v_3.1 mRNA with developmental age and the expression of Ca_v_3.2 and Ca_v_3.3 mRNAs peaked at P0 (figure 2*c*). These results are consistent with the expression of all three T-type Ca^2+^ channel isoforms throughout the period of development when TNFα reverse signalling enhances axon growth.

### 2.3. T-type Ca^2+^ currents are undetectable in superior cervical ganglion somata

**Figure 3.**
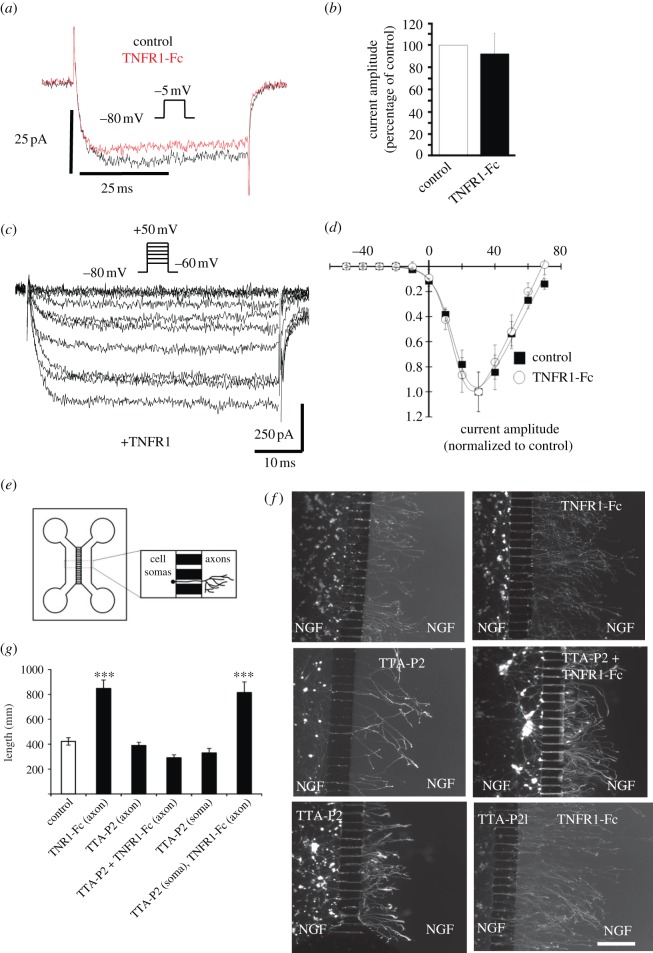
Functional significance of T-type Ca^2+^ channels in sympathetic axons. (*a*) Representative current traces resulting from a step potential from −80 mV to −5 mV before Fc fragment (control, black trace) and after 10–15 min application of TNFR1-Fc (5 µg ml^−1^; red trace). Ca^2+^ currents were recorded every 10 s before and after the application of TNFR1-Fc. Recordings were performed using 5 mM Ca^2+^ as a charge carrier. No clear difference in calcium current shape and amplitude is visible between the control and the treatment with TNFR1 suggesting that no T-type currents are functionally expressed in the somata of SCG neurons. (*b*) Bar chart shows the normalized peak Ca^2+^ current amplitude recorded from SCG neurons before (filled bar) and 10 to 15 min after starting TNFR1-Fc treatment (open bar, 92 ± 18%, *n* = 7 cells, *p* = 0.1328, paired *t*-test). (*c*) Representative current traces recorded from a TNFR1-treated SCG neuron in response to step potentials from −80 mV to between −60 mV to +50 mV in 10 mV increments. Recordings were performed using 10 mM Ca^2+^ as a charge carrier. (*d*) Normalized current–voltage relationship for calcium currents recorded from SCG neurons before (filled squares, *n* = 10 cells) and after 15 min TNFR1-Fc application (open circles, *n* = 6 cells). The mean ± s.e.m. data are fitted with a modified Boltzmann function with *V*_50,act_ of +17.4 ± 0.9 and +16.2 ± 0.5 mV, respectively, and normalized *G*_max_ of 2.5 ± 0.2 and 2.2 ± 0.2 nS pF^−1^, respectively. (*e*) Schematic illustration of the two-chamber microfluidic device. (*f*). Representative images of calcein-AM labelled P0 SCG neurons that were cultured for 24 h in a two-compartment microfluidic device containing 10 ng ml^−1^ NGF in both compartments, with or without 10 ng ml^−1^ TNFR1-Fc in the axon compartment and 200 nM TTA-P2 in either the axon or soma compartment, as indicated. Scale bar, 100 µm. (*g*) Bar chart of mean axon length of neurons projecting axons into the axon compartment under the experimental conditions indicated (control = NGF alone in both compartments). The data represent the mean ± s.e.m. of nine independent experiments (***p < 0.001, statistical comparison with control).

Given our evidence for the involvement of T-type Ca^2+^ channels in mediating the effects of TNFα reverse signalling on axon growth, we recorded Ca^2+^ currents from SCG neuron somata using whole cell patch clamp (figure 3). T-type channels are activated at low potentials (around –40 mV), and the current generated is characterized by a rapid activation and a rapid inactivation [23]. We initially recorded voltage-gated Ca^2+^ currents in response to a test potential to −5 mV from a holding potential of −80 mV using 5 mM Ca^2+^ as a charge carrier; in these conditions, T-type Ca^2+^ channels should be maximally activated with a reduced contribution of high voltage-activated Ca^2+^ current. Ca^2+^ currents were recorded for 5 min before applying TNFR1-Fc and then recorded every 10 s for 10–15 min (figure 3*a*). In control conditions, Ca^2+^ currents exhibited only a sustained, non-inactivating, component characteristic of high voltage-activated Ca^2+^ currents. After application of TNFR1-Fc, Ca^2+^ currents were similar in shape and amplitude (figure 3*a,b*). In order to maximize the size of the currents, we raised the Ca^2+^ concentration in the recording medium to 10 mM, and we compared the current–voltage relationships of the Ca^2+^ currents before and after treatment with TNFR1-Fc (figure 3*c,d*). In these conditions, no low voltage-activated transient Ca^2+^ current could be recorded after applying TNFR1-Fc and no difference in the current–voltage curves was recorded. Altogether, our results suggest that TNFα reverse signalling does not induce the functional expression of T-type channels in the somata of SCG neurons.

### 2.4. T-type Ca^2+^ channels are functionally relevant in sympathetic axons not in cell somata

The above results raise the possibility that T-type Ca^2+^ channels are only functionally relevant for axon growth enhancement by TNFα reverse signalling along the axons themselves. To test this, we blocked these channels in a compartment culture paradigm in which the cell somata and growing axons are cultured in different compartments separated by a barrier (figure 3*e*). We previously reported that addition of TNFR1-Fc to the axon compartment, but not to the soma compartment, enhances axon growth [9]. Here we studied the consequences of blocking T-type Ca^2+^ channels separately in the soma and axon compartments on TNFR1-Fc-enhanced axon growth. In these experiments, we seeded P0 SCG neurons into one compartment (the soma compartment) of a two-compartment device that contained NGF in both compartments to sustain neuronal survival and encourage axon growth from the soma compartment into the axon compartment. After a 24 h incubation, we labelled the axons in the axon compartment with the fluorescent vital dye calcein-AM, which also retrogradely labelled cell bodies of neurons that projected axons into the axon compartment. We used a stereological method to quantify the extent of axon growth in the axon compartment relative to the number of neurons projecting axons into this compartment. Addition of the T-type Ca^2+^ channel blocker TTA-P2 to either the axon compartment or the soma compartment had no significant effect on the extent of axon growth in the axon compartment in cultures that were not treated with TNFR1-Fc (figure 3*f,g*). Addition of TNFR1-Fc to the axon compartment significantly enhanced axon growth in this compartment. Enhanced axon growth was completely inhibited by the addition of TTA-P2 to the axon compartment, but not by TTA-P2 addition to the soma compartment (figure 3*f,g*). Similar experiments using the T-type Ca^2+^ channel blocker mibefradil yielded very similar results (not shown). These findings suggest that T-type Ca^2+^ channels are functionally relevant for axon growth enhancement by TNFα reverse signalling in axons, but not in somata.

### 2.5. TNFα reverse signalling induces Ca^2+^ transients in sympathetic axons via T-type Ca^2+^ channels

**Figure 4.**
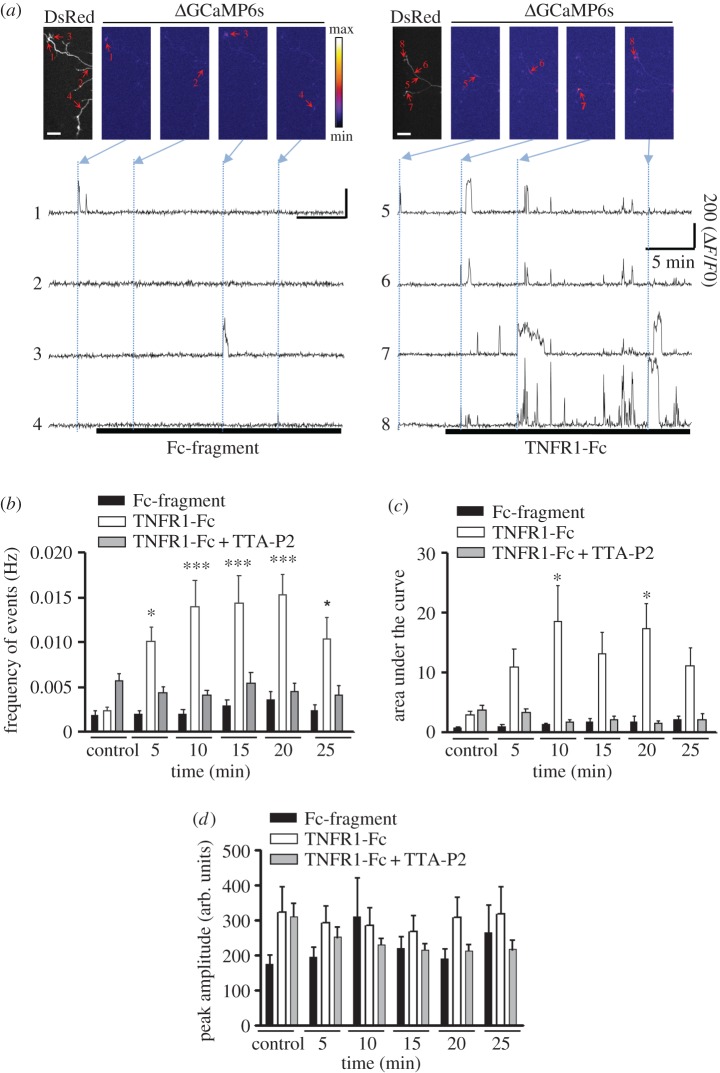
TNFR1-Fc promotes Ca^2+^ influx in sympathetic axons via T-type Ca^2+^ channels. (*a*) Micrographs of Ca^2+^ transients in neurites of SCG neurons co-transfected with DsRed and GCaMP6s-CAAX, grown with 10 ng ml^−1^ NGF and treated with either 5 µg ml^−1^ Fc fragment (left panels) or 5 µg ml^−1^ TNFR1-Fc (right panels). The black and white panels in the Fc fragment-treated and TNFR1-Fc-treated neurons show transfected neurons expressing DsRed, which was used to maintain focus during image acquisition. Red arrows indicate regions of interest in the axons (1, 2, 3 and 4 for Fc fragment-treated cells and 5, 6, 7 and 8 for TNFR1-Fc-treated cells). Scale bar, 50 µm. The pseudo colour scale between the panels indicates the GCaMP fluorescence intensity. Representative time courses of the GCaMP fluorescence signal corresponding to individual regions of interest in Fc fragment-treated (1–4) or TNFR1-Fc-treated (5–8) neurons. Δ*F*/*F*0 corresponds to the variation of fluorescence over the initial fluorescence and is expressed as an arbitrary unit. (*b*) Bar chart of the frequency of Ca^2+^ transients before and after application of either 5 µg ml^−1^ Fc fragment, 5 µg ml^−1^ TNFR1-Fc or TNFR1-Fc plus 1 µM TTA-P2. Mean ± s.e.m. of data derived from 143 regions of interest of 13 Fc-treated neurons, 235 regions of interest of 16 TNFR1-Fc-treated neurons and 216 regions of interest from 13 neurons treated with TNFR1-Fc plus TTA-P2 from three separate experiments of each type. *p < 0.05 TNFR1-Fc-treated versus control, ***p < 0.001 TNFR1-Fc-treated versus control, Bonferroni's multiple comparison test. (*c*) Bar chart of the area under individual Ca^2+^ peaks in the same experiments. (*d*) Bar chart of the peak amplitude in the same experiments.

To determine whether activation of TNFα reverse signalling induces Ca^2+^ transients in sympathetic axons, P0 SCG neurons were co-transfected with plasmids expressing mCherry or DsRed (to identify transfected neurons) and a genetically encoded Ca^2+^ sensor (GCaMP6s-CAAX) whose expression is targeted to the plasma membrane [24]. After transfection, the neurons were cultured for 12 h in medium containing 10 ng ml^−1^ NGF and 300 nM TAPI-O (TNFα processing inhibitor, which inhibits TACE and thereby maintains the level of membrane-integrated TNFα at the plasma membrane). Variations in fluorescence were then recorded in processes, and growth cones of SCG neurons perfused with a saline solution for 5 min before adding Fc fragment control protein or TNFR1-Fc (figure 4*a*). Prior to treatment with these reagents, there was a low frequency of Ca^2+^ transients. When analysed at 5 min intervals after the start of treatment, the signal frequency became significantly greater in TNFR1-Fc-treated cultures compared with Fc-treated control cultures between 5 and 10 min after the start of treatment, and remained significantly greater at each subsequent 5 min interval until the end of the recording period at 25 min (figure 4*b*). Interestingly, Ca^2+^ transients were local events and did not propagate to the rest of the neuron. These results suggest that TNFR1-Fc increases the frequency of Ca^2+^ transients. Integration of the areas under individual fluorescent peaks above a standard threshold revealed significant increases in TNFR1-Fc-treated cultures compared with Fc-treated cultures during each 5 min interval, starting at the 5–10 min interval (figure 4*c*). However, there was no significant increase in the amplitude of individual Ca^2+^ transient peaks in TNFR1-Fc-treated cultures compared with Fc-treated controls (figure 4*d*). Taken together, these results suggest that TNFR1-Fc not only increases the frequency of Ca^2+^ signals but also increases the duration of individual events. These significant increases in both the frequency and duration of fluorescent signals brought about by TNFR1-Fc were completely prevented by TTA-P2 (figure 4*b,c*), suggesting that these events were mediated by T-type Ca^2+^ channels. In addition to the temporal characteristics of fluorescence signals, differences were observed in their spatial distribution.

### 2.6. TNFR1-Fc-enhanced axon growth occurs independently of action potential generation

**Figure 5.**
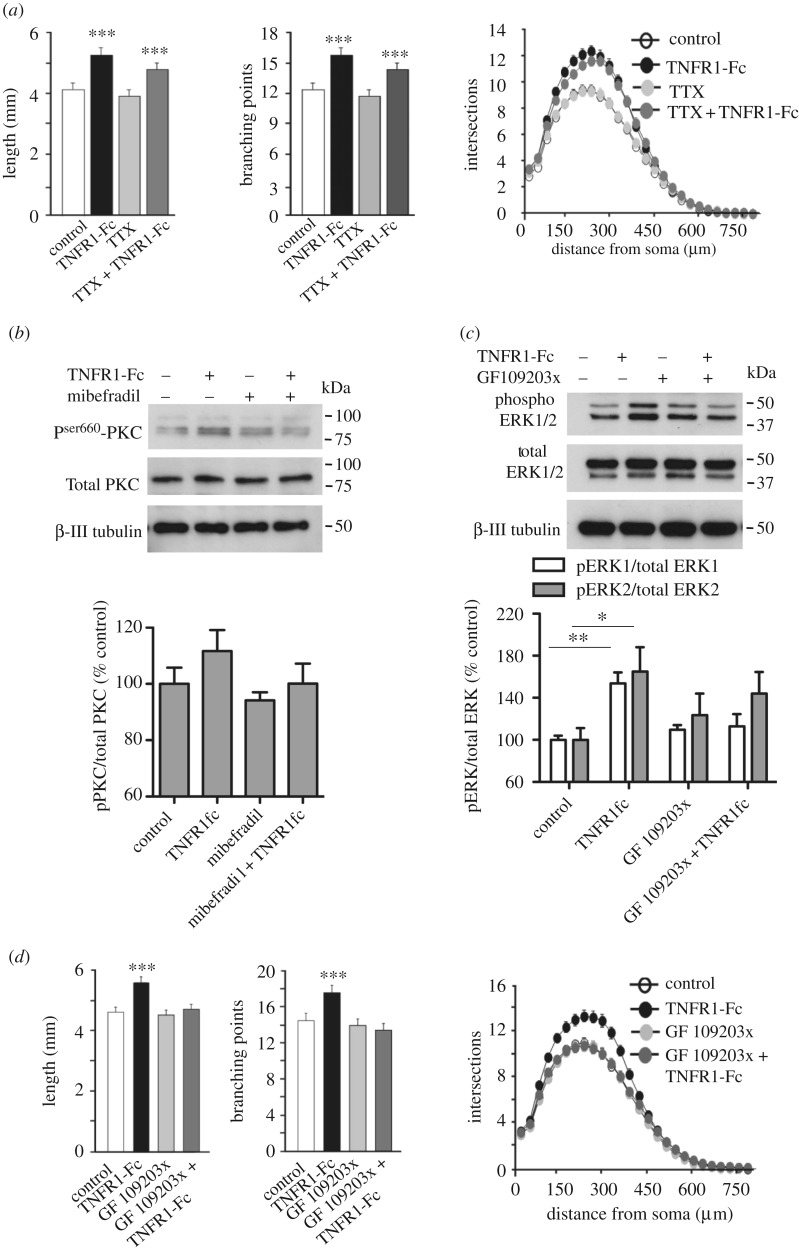
TNFR1-Fc-enhanced axon growth occurs independently of action potential generation and the requirement for PKC activation. (*a*) Bar charts of length and branch number and Sholl profiles of P0 SCG neurons cultured for 24 h with either 10 ng ml^−1^ NGF alone (control), NGF and 10 ng ml^−1^ TNFR1-Fc, NGF and 100 nM TTX or NGF, TNFR1-Fc and TTX. Mean ± s.e.m of neurite arbour data of 150 neurons per condition combined from three separate experiments of each type (***p < 0.001, statistical comparison with control). (*b*) Representative immunoblot probed for phospho-(Ser660) PKC, total PKC and β-III tubulin of lysates of P0 SCG neurons that were cultured for 12 h in medium containing 10 ng ml^−1^ NGF before being treated for 15 min with TNFR1-Fc and 1 µM mibefradil. The bar chart plots the densitometry from four separate western blots of the ratio of pPKC to total PKC, normalized to 100% for the controls, mean ± s.e.m. (*c*) Representative immunoblot probed for phospho-ERK1/phospho-ERK2, total ERK1/ERK2 and β-III tubulin of lysates of P0 SCG neurons that were cultured for 12 h in medium containing 10 ng ml^−1^ NGF before being treated for 15 min with 10 ng ml^−1^ TNFR1-Fc and 10 µM GF 109203X. The bar chart plots the densitometry from four separate western blots of the ratio of pERK1/2 to total ERK1/2, normalized to 100% for the controls (*p < 0.05 and **p < 0.01, statistical comparison with control). (*d*) Bar charts of length and branch point number and the Sholl profiles of P0 SCG neurons cultured for 24 h with either NGF alone (control), NGF and TNFR1-Fc, NGF and GF 109203X or NGF, TNFR1-Fc and GF 109203X. The data shown represent the mean ± s.e.m. of neurite arbour data of 150 neurons per condition combined from three separate experiments of each type (***p < 0.001, statistical comparison with control).

Because activation of T-type Ca^2+^ channels has been shown to trigger a burst of action potentials mediated by Na^+^ channels in many different kinds of neuron [23], we investigated whether action potential generation plays a role in TNFRl-Fc-promoted axon growth. To test this, we treated P0 SCG neurons with tetrodotoxin (TTX), a selective blocker of most voltage-gated Na^+^ channels and inhibitor of action potential generation in neurons. TTX did not inhibit TNFR1-Fc-promoted neurite growth, suggesting that the generation of action potentials is not required for enhanced axon growth (figure 5*a*). T-type channels are known to participate in low threshold oscillations in other tissues [25].

### 2.7. Protein kinase C activation is required for TNFR1-Fc-promoted axon growth

We have previously shown that TNFα reverse signalling enhances axon growth by activating MEK/ERK of the MAP kinase signalling cascade [9]. To elucidate the link between T-type Ca^2+^ channels, Ca^2+^ influx, elevation of [Ca^2+^]_i_ and MEK/ERK activation, we explored the potential role of PKC, a family of serine–threonine protein kinases that has been implicated in activating multiple signalling cascades, including MAP kinase signalling [26–28]. Of the 10 known PKC members, the conventional isozymes (PKC-α, PKC-βI, PKC-βII and PKC-γ) are activated by both [Ca^2+^]_i_ and diacylglycerol [29]. We began our investigation of the potential role of PKC in TNFR1-Fc-promoted axon growth by ascertaining whether TNFR1-Fc activates PKC in cultured SCG neurons. In these experiments, we first cultured P0 SCG neurons for 12 h with NGF before treating them with TNFR1-Fc. Western blot analysis revealed an increase in the level of phosphoserine 660 PKC-βII after 15 min exposure to TNFR1-Fc. This increase was prevented by preincubating the neurons with mibefradil (figure 5*b*).

To determine whether PKC activation is required for ERK1/ERK2 activation by TNFR1-Fc, we studied the effect of GF 109203X, a potent and selective inhibitor of PKC [30]. In these experiments, we first cultured P0 SCG neurons for 12 h with NGF before treating them with TNFR1-Fc. Western blot analysis revealed that the increases in phospho-ERK1 and phospho-ERK2 brought about by TNFR1-Fc treatment were partially or fully prevented by preincubating the neurons with GF 109203X, and that GF 109203X alone did not significantly affect the levels of phospho-ERK1 and phospho-ERK2 (figure 5*c*).

Finally, to investigate if PKC activation is required for TNFR1-Fc-promoted neurite growth, we examined whether GF 109203X could prevent this. In these experiments, we plated P0 SCG neurons in NGF-containing medium, pretreated them for 2 h with GF 109203X before adding TNFR1-Fc and imaging the neurite arbours 24 h later. GF 109203X prevented TNFR1-Fc enhanced neurite growth, as shown by quantification of neurite length, branch point number and Sholl analysis (figure 5*d*). Taken together, these findings suggest that PKC plays a role in mediating the effect of TNFα reverse signalling on axon growth.

## 3. Discussion

The data reported here establish the essential role of T-type Ca^2+^ channels in mediating the effects of TNFα reverse signalling on axon growth in developing sympathetic neurons. We show that each of three selective T-type Ca^2+^ channel inhibitors, mibefradil, TTA-A2 and TTA-P2, completely prevent TNFR1-Fc-promoted axon growth without affecting axon growth in the absence of TNFR1-Fc or affecting neuronal survival. In contrast, blockers of L-type, N-type, P/Q-type and R-type Ca^2+^ channels have no effect on TNFR1-Fc-promoted axon growth. Furthermore, we show that TNFR1-Fc, but not Fc control protein, significantly increases the frequency and duration of Ca^2+^ transients recorded using a genetically encoded Ca^2+^ sensor targeted to the plasma membrane, and that pharmacological blockade of T-type Ca^2+^ channels completely prevents these increases. Given the importance of Ca^2+^ influx and subsequent elevation of intracellular Ca^2+^ to TNFR1-Fc-promoted axon growth [9], our findings show that Ca^2+^ influx triggered by activation of TNFα reverse signalling is mediated by T-type Ca^2+^ channels and further support the crucial role of T-type Ca^2+^ channels in TNFR1-Fc-promoted axon growth.

Our compartment culture results suggest that T-type channels are only functionally relevant in axons for TNFR1-promoted growth. TNFR1 only promoted axon growth when applied to axons not somata and T-type Ca^2+^ channel inhibitors only inhibited TNFR1-promoted axon growth when applied to axons not somata. These results are consistent with our whole-cell patch-clamp studies which did not detect T-type Ca^2+^ currents at the somata. The absence of T-type Ca^2+^ currents in the somata of sympathetic neurons, as also shown in rat and frog SCG neurons [31–33], implies that T-type Ca^2+^ channels are not expressed in this location in sufficient numbers to be detected. T-type calcium channels have only been identified in the cell bodies of a small subset of sympathetic neurons [33]. This could be because these Ca^2+^ channels migrate into axons as the neurons extend axons in culture or because Ca^2+^ channels are synthesized locally in axons because the encoding mRNA is transported along axons prior to translation [34]. However, they were not previously detected in developing axons or growth cones of frog superior cervical neurons [33]. We detected transcripts encoding all three T-type Ca^2+^ channel isoforms in SCG throughout the period of development when TNFα reverse signalling enhances axon growth. This raises the possibility that all three subtypes could contribute to axon growth, in the presence of TNFR1. Because subtype-specific T-type Ca^2+^ channel inhibitors are not currently available, we were unable to determine pharmacologically whether a particular subtype plays a more prominent role than others. While various anti-T-type channel antibodies have been reported, they have questionable specificity for different T-type Ca^2+^ channel isoforms and are thus of limited use for immunocytochemical studies of the distribution of these channel proteins.

Our imaging studies show that TNFα reverse signalling modulates Ca^2+^ signalling by affecting the frequency and duration of discrete events. TNFR1-Fc causes highly significant increases in the frequency and duration of intracellular Ca^2+^ transients and these increases are prevented from occurring by T-type Ca^2+^ channel blockers. However, the T-type channel blocker did not abolish these events under control conditions, indicating that other Ca^2+^ channels are also involved in these Ca^2+^ transients, and opening the possibility that T-type channels were either inserted in the membrane by TNFR1-Fc, or it caused a shift in the window current for T-type channels [23], resulting in their increased availability at the resting membrane potential in the axons.

An interesting issue is how TNFα reverse signalling influences the opening of T-type channels. It is well documented that T-type Ca^2+^ channels open secondary to small membrane depolarizations [23,35]. Thus, it is possible that TNFα reverse signalling causes T-type Ca^2+^ channel opening by first causing membrane depolarization. However, our current clamp studies did not provide any evidence in support of this in the cell body (data not shown). This raises the possibility that TNFα reverse signalling only affects the membrane potential in the developing sympathetic axons, inducing oscillations, or affects T-type channels by another means, for example by changing the voltage-dependence of the window current, which is maximal at about −60 mV in 2 mM Ca^2+^ [36]. There is evidence of numerous post-translational modifications of channels that affect T-type channel properties [37,38].

In this study, we have demonstrated the crucial role of T-type Ca^2+^ channels in mediating the effects of TNFα reverse signalling on sympathetic axon growth. TNFα reverse signalling has also been reported to enhance the growth of sensory axons [39], which raises the question of whether T-type Ca^2+^ channels play a role in sensory axon growth. In addition to the effects of TNFα reverse signalling on axon growth, an additional example of reverse signalling within the TNF superfamily affecting axon growth has recently been reported. CD40 ligand (CD40L, TNFSF5) reverse signalling in subsets of sympathetic neurons enhances axon growth, and this is required *in vivo* for the ramification of axons in the tissues innervated by these neurons [40]. Again, it would be of interest to determine whether T-type Ca^2+^ channels and Ca^2+^ influx play any role in mediating CD40 L reverse signalling in developing sensory neurons, where T-type channels have been identified in the somata of specific subtypes [41,42], and play important physiological and pathological roles [43].

We have previously shown that ERK1/ERK2 is activated in developing sympathetic neurons by TNFR1-Fc and that pharmacological inhibition of ERK1/ERK2 activation prevents the axon growth response to TNFR1-Fc [9]. To ascertain the link between TNFR1-Fc-promoted T-type Ca^2+^ channel opening and ERK activation, we explored the potential role of PKC, which is activated by Ca^2+^ signals and activates in turn a variety of signalling pathways including ERK [29]. We showed that TNFR1-Fc enhanced PKC activation and that pharmacological inhibition of PKC reduced ERK1/ERK2 activation by TNFR1-Fc. Importantly, pharmacological inhibition of PKC completely prevented the axon growth response of sympathetic neurons to TNFR1-Fc. Taken together, our findings have established a sequence of essential steps that link TNFα reverse signalling with enhanced axon growth in developing sympathetic neurons. Activation of TNFα reverse signalling enhances intracellular Ca^2+^ transients mediated by T-type Ca^2+^ channels in axons, leading to activation of PKC and activation of ERK1/ERK2. In future work, it will be particularly interesting to establish how TNFα reverse signalling modulates the opening of T-type Ca^2+^ channels, initiating this chain of events.

## 4. Conclusion

We have discovered a novel and unexpected function for voltage-gated T-type Ca^2+^ channels in axons of SCG sympathetic neurons, which do not exhibit T-type channels in their somata. We show that these channels mediate Ca^2+^ transients in developing sympathetic axons following activation of TNF reverse signalling and that they are essential for the enhanced axon growth and branching promoted by TNF reverse signalling. Given the physiological significance of TNF reverse signalling for establishing sympathetic innervation, this constitutes a clear role for voltage-gated T-type Ca^2+^ channels in sympathetic neuron development.

## 5. Material and methods

### 5.1. Neuron culture

Dissected SCG were freed of adherent connective tissue using tungsten needles and were trypsinized and plated at very low density (approx. 200 neurons per dish/well) in polyornithine and laminin-coated 35 mm tissue culture dishes (Greiner, Gloucestershire, UK) or four-well dishes (Starlab, Milton Keynes, UK) in serum-free Hams F14 medium [44] supplemented with 0.25% Albumax I (Invitrogen, Paisley, UK). Neuronal survival was estimated by counting the number of neurons in four-well dishes 2 h after plating and again at 24 h. All neurons in each well were counted. The number of neurons surviving at 24 h was expressed as a percentage of the initial number of neurons counted. Analysis of the size and complexity of neurite arbours was carried out in 35 mm dishes 24 h after plating. The neurite arbours were labelled by incubating the neurons with the fluorescent vital dye calcein-AM (1 : 1000, Invitrogen, Paisley, UK) at the end of the experiment. Images of neurite arbours were acquired by fluorescence microscopy and analysed to obtain total neurite length, number of branch points and Sholl profiles [45].

For studying the effects of regional blockade of T-type Ca^2+^ channels on neurite growth, P0 SCG neurons were plated in one compartment of a two-compartment microfluidic device (Xona microfludics, CA, USA). Both compartments received NGF and the TNFR1-Fc was added to the axon compartment. A T-type Ca^2+^ channel blocker was added to either the soma or axon compartment. After 24 h incubation, the axons in the axon compartment and the cell bodies that projected axons into this compartment were labelled by adding the fluorescent vital dye calcein-AM to the axon compartment. Axon length was quantified by a modification of a previously described method [46]. Briefly, using NIH ImageJ, a grid of vertical lines was traced with an interline interval of 200 µm. Total intersections between neurites and the grid were counted and normalized against the number of labelled somas in the cell body compartment. Average neurite length per projecting cell body was calculated using the formula *L* = *πDI*/2, where *L* is the estimated length, *D* is the interline interval and *I* the average number of intersections per projecting cell body. Measurements were independently carried out in all fields along the microfluidic barrier.

Purified recombinant NGF and TNFR1-Fc and caspase inhibitor Q-VD-OPh were obtained from R&D Systems. Dotarizine, ω-agatoxin TK, ω-grammotoxin SIA and mibefradil were obtained from Santa Cruz Biotechnologies, Heidelberg, Germany. Nifedipine was obtained from Calbiochem, Watford, UK. SNX 482 and GF 109203x were obtained from Tocris Biosciences, Abingdon, UK. TTA-A2 and TTA-P2 were obtained from Alomone, Jerusalem, Israel. TTX was obtained from Abcam, Cambridge, UK. TAPI-O was obtained from Enzo Life Sciences, Exeter, UK.

### 5.2. GCaMP imaging in superior cervical ganglion neurons

SCG neurons were co-transfected with either 0.8 µg pCAGGs-mCherry (Addgene) or 0.8 µg pDsRed-Express-N1 (Clontech) and 2.5 µg membrane-directed pGPCMV-GCaMP6s-CAAX (Addgene) using a microporator (Digital Bio; 2 × 30 ms pulse at 900 V) and cultured at a high density in medium containing 10 ng ml^−1^ NGF and 300 nM TAPI-O on glass coverslips that had been treated with polyornithine and laminin. After 24 h, the coverslips were mounted in a laminar-flow perfusion and stimulation chamber (Warner Instruments) on the stage of an epifluorescence microscope (Axiovert 200M, Zeiss). White and 470 nm LEDs served as light sources (Cairn Research, UK). Fluorescence excitation was done through an X20 0.75 NA Fluar Zeiss objective using 470/40 nm and 572/35 nm excitation and 59022bs dichroic filters (Chroma). Simultaneous acquisition of GCaMP and mCherry/DsRed was performed using an OptoSplit II (Cairn Research, UK) with 565 nm dichroic and 520/40 nm and 632/60 nm emission filters (Chroma). GCaMP6 and mCherry/DsRed fluorescence was collected at 1 Hz with an Andor iXon+ (model DU-897U-CS0-BV) back-illuminated EMCCD camera using OptoMorph software (Cairn Research, UK). An OptoMask (Cairn Research, UK) was used to acquire two 512 × 256 pixel recordings of the same field of view (one for GCaMP6 and one for mCherry/DsRed). Cells were perfused (0.5 ml min^−1^) in a saline solution at 35°C containing 119 mM NaCl, 2.5 mM KCl, 2 mM CaCl_2_, 2 mM MgCl_2_, 25 mM HEPES (buffered to pH 7.4) and 30 mM glucose for 5 min before the addition of either 5 µg ml^−1^ TNFR1-Fc or 5 µg ml^−1^ human Fc-fragment or 5 µg ml^−1^ TNFR1-Fc + 1 µM TTA-P2 for a further 25 min. The last condition had an additional initial perfusion of 5 min with 1 µM TTA-P2 alone. Transfected neurons were initially identified by stimulating the preparation at 33 Hz for 180 ms every 4 s (1 ms current pulses via platinum electrodes). Analysis was performed with ImageJ (http://rsb.info.nih.gov/ij), using a custom-written plugin (http://rsb.info.nih.gov/ij/plugins/time-series.html). Briefly, regions of interest (ROI, 4 µm diameter circles) in the processes of neurons were selected where rapid increases in GCaMP6 fluorescence occurred. Using the same threshold applied to all ROI, the frequency of peaks above the threshold were calculated for each ROI, followed by the average peak amplitude and the average area under the curve of the peaks.

### 5.3. Electrophysiological recordings

High density (50 000 neurons per 35 mm dish) SCG neuronal cultures were used for electrophysiological experiments after 24 h in culture in medium containing 10 ng ml^−1^ NGF and 300 nM TAPI-O (Enzo Life Sciences). Whole-cell patch-clamp recordings were performed at room temperature (21–25°C) before and after the addition of 5 µg ml^−1^ TNFR1-Fc or 5 µg ml^−1^ human Fc fragment (Abcam) for 10–15 min. Single cells were voltage clamped using an Axopatch 200B patch-clamp amplifier (Axon instruments). Patch pipettes were filled with a solution containing the following (in mM): 140 Cs-aspartate, 5 EGTA, 2 MgCl_2_, 0.1 CaCl_2_, 2 K_2_ATP and 10 HEPES, titrated to pH 7.2 with CsOH. The external solution contained the following: 150 mM tetraethylammonium bromide, 3 mM KCl, 1 mM NaHCO_3_, 1 mM MgCl_2_, 10 mM HEPES, 4 mM glucose and 5 or 10 mM CaCl_2_, pH adjusted to 7.4 with Tris base. Measurement and analysis were performed as previously described [47]. Normalized current–voltage relationships were fitted with a modified Boltzmann equation as follows: *I* = *G*_max_ × (*V* − *V*_rev_)/(1 + exp( − (*V* − *V*_50,act_)/*k*)), where *I* is the current density (in picoamperes × picofarad^−1^), *G*_max_ is the maximum conductance (in nanosiemens × picofarad^−1^), *V*_rev_ is the reversal potential in mV, *V*_50,act_ is the midpoint voltage for current activation in mV, and *k* is the slope factor.

### 5.4. Real-time QPCR

The levels of Cav3.1, Cav3.2 and Cav3.3 mRNAs were quantified by RT-QPCR relative to a geometric mean of mRNAs for the housekeeping enzymes glyceraldehyde phosphate dehydrogenase (GAPDH), succinate dehydrogenase (SDHA) and hypoxanthine phosphoribosyltransferase 1 (HPRT1). Total RNA was extracted from whole SCG with the RNeasy Mini extraction kit (Qiagen, Crawely, UK), and 5 µl was reverse transcribed for 1 h at 45°C using the AffinityScript kit (Agilent, Berkshire, UK) in a 25 µl reaction according to the manufacturer's instructions. cDNA (2 µl) was amplified in a 20 µl reaction volume using Brilliant III ultrafast QPCR master mix reagents (Agilent, Berkshire, UK). QPCR products were detected using dual-labelled (FAM/BHQ1) hybridization probes specific to each of the cDNAs (MWG/Eurofins, Ebersberg, Germany). The PCR primers were: *gapdh* forward 5′-GAG AAA CCT GCC AAG TAT G-3′ and reverse 5′-GGA GTT GCT GTT GAA GTC-3′; *sdha* forward 5′-GGA ACA CTC CAA AAA CAG-3′ and reverse 5′-CCA CAG CAT CAA ATT CAT-3′; *hprt1* forward 5′-TTA AGC AGT ACA GCC CCA AAA TG-3′ and reverse 5′-AAG TCT GGC CTG TAT CCA ACA C-3′; *Cav3.1* forward 5′-CTG GTT ATT CTC CTC AAC T-3′ and reverse 5′-TTC CCA AAG ATA CCC AAA-3′; *Cav3.2* forward 5′-TGC TTC TTC GTC TTC TTC-3′ and reverse 5′-CAG ATG AAT GGG TTC TCC-3′; *Cav3.3* forward 5′-CAT TGG AAA CAT TGT CCT C-3′ and reverse 5′-CAG TGA TAG AAC TTG CCT-3′. Dual labelled probes were: *gapdh*, FAM-AGA CAA CCT GGT CCT CAG TGT-BHQ1; *sdha*, FAM-CCT GCG GCT TTC ACT TCT CT-BHQ1; *Hprt1*, FAM-TCG AGA GGT CCT TTT CAC CAG CAA G-BHQ1; *Cav 3.1*, FAM-CGA CCA TCT TCA CCA CCA-BHQ1; *Cav3.2*, FAM-CCT CCT CTG TCT GGT AGT ATG GC-BHQ1 and *Cav3.3*, FAM-CGC CTT CTT CAT CAT CTT CGG T-BHQ1. Forward and reverse primers were used at a concentration of 150 nM each and dual-labelled probes were used at a concentration of 300 nM. PCR was performed using the Mx3000P platform (Agilent, Berkshire, UK) using the following conditions: 45 cycles of 95°C for 12 s and 60°C for 35 s. Standard curves were generated in every 96-well plate, for each cDNA for every real-time PCR run, by using serial threefold dilutions of reverse transcribed spleen total RNA (Ambion, Paisley, UK). Three separate dissections were performed for each age.

### 5.5. Immunoblotting

For harvesting protein for western blot, neurons were cultured at a high density (approx. 85 000 neurons per well) in 96-well plates. Immunoblotting was carried out using the BioRad TransBlot (BioRad, Hertfordshire, UK) as previously described [48]. The blots were probed with antibodies to phospho-PKC (pan βII Ser660, 1 : 1000, Cell Signaling, Hertfordshire, UK, catalogue number 9371), PKC (Millipore, Watford, UK, catalogue number 05-983), phospho-ERK1/ERK2 (1 : 1000, Cell Signaling, catalogue number 9101), total ERK1/ERK2 (1 : 1000, Cell Signaling, catalogue number 9102), β-III tubulin (1 : 10 000, R&D systems, Abingdon, UK, catalogue number MAB119). Binding of the primary antibodies was visualized with an HRP-conjugated secondary antibody (1 : 2000; Promega, Southampton, UK) and ECL-plus (Amersham, Buckinghamshire, UK). All primary antibodies labelled bands of the expected sizes.

### 5.6. Statistical analysis

Statistical comparisons were performed by independent Student's *t*-test or one-way ANOVA followed by Fisher's *post hoc* test.
